# Asthma control and management among schoolchildren in urban Uganda: results from a cross-sectional study

**DOI:** 10.12688/wellcomeopenres.15460.1

**Published:** 2019-11-05

**Authors:** Harriet Mpairwe, Pius Tumwesige, Milly Namutebi, Marble Nnaluwooza, Tonny Katongole, Josephine Tumusiime, Barbara Apule, Caroline Onen, Mike Mukasa, Joseph Kahwa, Emily L. Webb, Neil Pearce, Alison M. Elliott

**Affiliations:** 1 MRC/UVRI and LSHTM Uganda Research Unit, Plot 51-59 Nakiwogo Road, Box 49, Entebbe, Uganda; 2 London School of Hygiene & Tropical Medicine, Keppel St, Bloomsbury, London, WC1E 7HT, UK

**Keywords:** Asthma control, asthma management, schoolchildren, Uganda, salbutamol, inhaled corticosteroids, Asthma Control Test

## Abstract

**Background**: Children from low- and middle-income countries have poor asthma control, mainly because of poor management. The extent of this problem in Uganda is not well known, but such information would be useful to guide policy and practice. We therefore conducted a cross-sectional study among schoolchildren with asthma in urban Uganda, to assess the level of asthma control and management.

**Methods**: Schoolchildren aged 5-17 years were enrolled, asthma was diagnosed by the study medical team. Asthma control was assessed using the Asthma Control Test and the childhood Asthma Control Test. Data on previous asthma management was obtained using interviewer-led questionnaires. Data were analysed using multiple linear and multiple logistic regression.

**Results**: We enrolled 561 children with asthma, of whom only 56% had ever had an asthma diagnosis. We categorised asthma as well-controlled (55.5%), partly-controlled (29.5%) and poorly-controlled (15.0%). Poor asthma control was associated with increasing age (adjusted regression coefficient [95% confidence interval], p-value: -1.07 [-1.20, -0.94], p<0.0001), concurrent allergic rhinitis (-1.33 [-2.28, -0.38], p=0.006), and city residence in early life (-1.99 [-3.69, -0.29], p=0.06). Regular use of inhaled asthma medication in the last 12 months was very low; 18.1% for salbutamol and 6.7% for inhaled corticosteroids. The main barriers to inhaled asthma medication use were lack of prescription (47.6%) and inaccurate diagnosis (38.8%). Increased inhaler use was associated with tertiary education of the fathers (adjusted odds ratio [95% confidence interval], p-value: 5.19 [2.39-11.28], p<0.0001), city residence in early life (4.66 [1.79-12.43], 0.002) and an asthma diagnosis prior to enrolment (11.39 [6.35-20.43], p<0.0001).

**Conclusions**: This study confirms that children with asthma in Uganda generally have inadequate asthma control, which is attributable to poor asthma management. This could be improved through re-training of medical workers and patient education, and by increasing availability and affordability of essential asthma medications.

## Background

Poor asthma control among children in low- and middle-income countries (LMICs) is believed to be relatively common, and mainly because of poor asthma management. Studies have shown increased asthma severity among children in LMICs compared to children in high income countries (HICs) ^1^. The health systems in most LMICs are not well adapted to the management of chronic conditions ^2^. In several LMICs, essential asthma medications, such as inhaled bronchodilators (salbutamol) and inhaled corticosteroids, are not on the country’s essential drugs lists ^3^ and are not easily available ^4^. This leads to poor asthma control, higher rates of asthma attacks and higher mortality ^5^.

The level of asthma control and management among children from the general population has not been investigated in Uganda, and there have been few investigations in other low income countries. This information is important in informing policy on the extent of the problem, and in identifying areas where improvements could be made cost-effectively. We therefore conducted a cross-sectional study among schoolchildren in urban Uganda with asthma, to assess their levels of asthma control and previous management.

## Methods

### Study design

This cross-sectional study included all schoolchildren with asthma who participated in a large case-control study whose aim was to investigate the risk factors for asthma ^6^. We report using the STROBE guidelines ^7^.

### Study population, recruitment and consent

We recruited schoolchildren, 5–17 years, from primary and secondary schools in an urban area of Wakiso District, in central Uganda. At each school, all children were pre-screened for breathing problems by the study nurses; this involved requesting all children with any current breathing problems to register with the study team or the class teacher. Children with any self-reported breathing problems were provided with cards inviting their parents or guardians for a parents’ meeting. During the meeting, the study team explained what the study was about, talked about asthma in general and responded to any questions or concerns that the parents raised. Parents or guardians interested in their child participating in the study provided written informed consent for participation in the study and publication of anonymised study findings. The consent process was conducted in either English or Luganda (the main local language). Children eight years or older provided written informed assent. Study enrolment was between May 2015 and July 2017.

### Ethical approval

The study was approved by the Uganda Virus Research Institute Research and Ethics Committee (reference number: GC/127/14/09/481), and the Uganda National Council for Science and Technology (reference number: HS 1707).

### Asthma diagnosis

All study procedures were conducted at the school premises, to minimise interruption of the pupils’ studies. We screened for asthma with the International Study of Asthma and Allergies in Childhood (ISAAC) questionnaire ^8^. Children with wheezing in the last 12 months were further assessed by the study medical or clinical officers, including a detailed medical and treatment history reported by parents or adolescents and clinical assessments including spirometry. Asthma diagnosis was mostly clinical, as a history of recurrent symptoms of wheezing, chest tightness, shortness of breath and cough (that is mostly dry, worse at night and in the morning). In addition, we assessed for forced expiratory volume in the first second (FEV _1_) and considered values <80% of the expected values for age, sex, height and race abnormal. However, this contributed to, but was not a requirement for, the asthma diagnosis. We enquired, from the parents or the adolescents, about medical history of a prior physician asthma diagnosis and good response to asthma medication. For children whose asthma diagnosis was not straightforward, two clinicians reviewed that participant and if they disagreed, that participant was excluded from the study. Children who did not report asthma symptoms in the last 12 months were also excluded from this study.

We collected data on asthma symptoms in the last four weeks using the childhood Asthma Control Test (c-ACT) ^9^ for children less than 12 years, and the Asthma Control Test (ACT) for children 13 years and older ^10^. We enquired about asthma medications that the children had ever used in the past, as well as medications that they had used regularly because of asthma symptoms in the last 12 months. Questionnaires ^11^ were interviewer-led and were answered by either the adolescents or by the parents/guardians for the younger children.

The clinical assessments included spirometry using a handheld device (Micro 1 Diagnostic Spirometer, CareFusion, Chatham Marine, United Kingdom); skin prick tests (SPT) with seven allergen extracts ( *Dermatophagoides* mix [ *D. farinae* and *D. pteronyssminus*], *Blomia tropicalis*, *Blattella germanica*, peanut, cat, pollen mix [weeds], and mould mix [ *Aspergillus* mix], with negative saline and positive histamine controls [ALK Abello, Hoersholm, Denmark]), using standard procedures described previously ^12^. This involved pricking through droplets of the allergens on the volar aspect of the participant’s arm with a skin lancet; the size of the wheal was measured after 15 minutes. Fractional exhaled nitric oxide (FENO) was measured using a handheld device (NoBreath ^®^ from Bedfonf Scientific, Maidstone, United Kingdom).

### Data management and statistical methods

Data were collected using pre-coded questionnaires ^11^ and double entered into OpenClinica open source software version 3.1.4 (OpenClinica LLC and collaborators, Waltham, MA, USA), and analysed in STATA, version 15 (StataCorp, Texas, USA).

For analyses where the outcome was asthma control scores as a continuous variable, the independent t-test or one-way ANOVA were used for univariate analysis, and multiple linear regression was used to control for confounders. For binary outcomes, we used standard chi square tests and multiple logistic regression. We built the final multiple regression models by adding one potential confounder at a time and examined the change in the main effect estimate; factors that were closely related (such as father’s and mother’s education level, or area of residence at birth and in the first five years) were not included in the same model to avoid multicollinearity ^13^. We did not impute for missing data.

## Results

### Characteristics of study participants

We enrolled 562 children with asthma from 55 schools, but one child was excluded from the current analysis because of incomplete data. Detailed participant flow diagram is published ^6^. At enrolment, only four children had wheezing symptoms; and of 477 (85%) children who successfully underwent spirometry, only three had forced expiratory volume in the first second (FEV _1_) values less than 80% of predicted values ^14^. The mean age was 11.4 years (range 5–17 years), 52.8% were girls and 47.3% and 37.9% had fathers and mothers with tertiary education, respectively ( Table 1). Based on the ISAAC questionnaire, 28.7% children reported four or more wheezing attacks in the last 12 months; 71.9% reported that their chest sounded wheezy during or after exercise in the last 12 months; 74.0% reported a dry cough at night in the last 12 months that was not associated with a common cold or chest infection; however, only 55.9% had ever had an asthma diagnosis ( Table 1).

**Table 1.** Characteristics of the Ugandan schoolchildren with asthma (N=561).

Characteristic	n	%
**Age** (years) mean, range (m=1)	11.4	5 - 17
**Sex**
Boys	265	47.2
Girls	296	52.8
**Father’s highest education level attained** (m=5)		
None/Primary	99	17.8
Secondary	194	34.9
Tertiary	263	47.3
**Mother’s highest education level attained** (m=7)		
None/Primary	159	28.7
Secondary	185	33.4
Tertiary	210	37.9
**Number of wheezing attacks in the last 12 months** ^‡^ (m=4)		
One	150	26.9
2–3	247	44.4
4 or more	160	28.7
**Ever had asthma diagnosis** (m=8) ^‡^		
Yes	309	55.9
**Chest sounded wheezy during or after exercise** (m=9) ^‡^		
Yes	397	71.9
**Had dry cough at night not associated with a cold, ‘flu’** **or chest infection** (m=8) ^‡^		
Yes	409	74.0
**Asthma control in the last four weeks, based on ACT** **and cACT scores** (m=8)		
Well controlled (scores >19)	307	55.5
Partly controlled (scores15–19)	163	29.5
Poorly controlled (scores <15)	83	15.0

N, number; m, missing; ^‡^Based on the International Study of Asthma and Allergies in Childhood (ISAAC) questionnaire; ACT, Asthma Control Test; cACT, childhood Asthma Control Test.

### Factors associated with asthma control

Total scores from the ACT and c-ACT were generated for each child, using the standard cut-off points of >19 for well-controlled, 15–19 for partly controlled, and <15 for poorly controlled asthma. Asthma control categories were 55.5% well-controlled, 29.5% partly controlled and 15.0% poorly controlled ( Table 1).

We assessed factors associated with asthma control, based on ACT and c-ACT test scores (low scores indicate poorer asthma control). We found that older children were more likely to have lower asthma control scores (adjusted coefficient [95% confidence interval], p-value: -1.07 [-1.20, -0.94], p<0.0001); children who reported regular vigorous physical activity three or more times a week (WHO recommendation ^15^) had higher scores (0.84 [0.01, 1.67], p=0.05); children with concurrent allergic rhinitis had lower scores (-1.33 [-2.28, -0.38], p=0.006); children who reported receiving antimalarial medication twice or more in the last 12 months had lower scores (-1.38 [-2.31, -0.45], p=0.007); and children who spent most of their first five years in the city had lower scores (-1.99 [-3.69, -0.29], p=0.06) ( Table 2). However, SPT and FENO were not significantly associated with asthma control.

**Table 2.** Factors associated with asthma control test scores among Ugandan schoolchildren.

Participants’ characteristics	Asthma control test scores ^#^ N=553			
	Univariate analysis		Multivariate analysis ^‡^	
	Mean (SD)	p-value	Adjusted mean difference (95% CI)	p-value
**Age** [m=1]				
5–12 years (n=338)	27.32 (3.80)		ref	
13–17 years (214)	18.37 (4.60)	<0.0001	-1.07 (-1.20, -0.94)	<0.0001
**Sex**				
Male (261)	25.18 (5.46)		ref	
Female (292)	22.68 (6.23)	<0.0001	-0.54 (-1.34, 0.26)	0.18
**Regular physical exercise as recommended by WHO**				
No (251)	21.91 (6.08)		ref	
Yes (302)	25.49 (5.44)	<0.0001	0.84 (0.01, 1.67)	0.05
**Concurrent allergic rhinitis** [m=1]				
No (434)	24.40 (5.97)		ref	
Yes (118)	21.88 (5.77)	0.0001	-1.33 (-2.28, -0.38)	0.006
**Received antimalarial treatment in the last 12 months** [m=1]				
None (208)	24.45 (5.89)		ref	
Once (169)	24.56 (5.76)		-0.15 (-1.08, 0.78)	
Twice or more (175)	22.48 (6.19)	0.001	-1.38 (-2.31, -0.45)	0.007
**Area of residence in first five years of life**				
Rural (71)	23.62 (5.18)		ref	
Town (433)	24.18 (6.09)		-0.60 (-1.77, 0.57)	
City (49)	21.37 (5.86)	0.007	-1.99 (-3.69, -0.29)	0.06
**Skin prick test responses** [m=9]				
Negative (<3mm) (244)	24.00		ref	
Positive (≥3mm) (300)	23.75	0.63	-0.51 (-1.31, 0.29)	0.21
**Fractional exhaled nitric oxide levels** [m=13]				
Normal (<35ppb) (335)	23.91		ref	
Elevated(≥35ppb) (195)	23.62	0.59	0.42 (-0.39, 1.24)	0.31

N, number; SD, standard deviation; CI, confidence interval; ref, reference; m, missing; WHO, World Health Organisation; ppb, parts per billion. ^#^Asthma control test scores are based on Asthma Control Test (ACT) and childhood Asthma Control Test (cACT); scores ranged from 8–34, low scores indicating poor asthma control and high scores indicating well controlled asthma. ^‡^Final linear regression model adjusted for age, sex, exercise as recommended by WHO, concomitant allergic rhinitis, antimalarial treatment, area of residence in early life and father’s education level; (model constant =37.30, R ^2^=0.44, F-ratio [10, 537] =41.69, p<0.0001, N=548).

### Reported medications previously used for asthma

Only 26.8% (148/553) children reported having ever used inhaled asthma medications, and 29.7% (164/553) reported having ever used herbal remedies for asthma management. Participants reported that the medications they used regularly (for asthma symptoms) in the last 12 months were steroid tablets (particularly prednisolone, 27.0%), salbutamol inhaler (18.1%), salbutamol tablets (17.4%), inhaled corticosteroids (6.7%), antihistamines (particularly cetirizine, 6.2%), aminophylline tablets or injections (5%), unspecified cough remedies (4.6%), antibiotics (2.8%), other unspecified tablets (2.6%), herbal remedies (2.5%), and nebulised salbutamol (1%).

We looked at the correlation between asthma control in the last four weeks and type of asthma medication used in the last 12 months. This was to assess the proportion of children currently with poor asthma control who had received the correct asthma treatment at any point in the last 12 months. We found that among 83 children currently with poorly controlled asthma, only 19 (20.9%) and six (7.2%) had used inhaled salbutamol and inhaled corticosteroids, respectively, in the last 12 months ( Table 3). Instead, a larger proportion had regularly used salbutamol tablets (26.5%) and steroid tablets (28.9%). We observed that of the 307 children with *well-controlled* asthma, 153 (49.8%) reported not using salbutamol or steroids in any formulation ( Table 3), suggesting that perhaps they had mild asthma.

**Table 3.** Asthma control in last four weeks and regularly used asthma medications in the last 12 months (N=553).

Regular asthma medication in the last 12 months	Asthma control in the last four weeks (ACT and cACT scores)			
	Well controlled, n (%) (scores >19) N=307	Partly controlled, n (%) (scores 15–19) N=163	Poorly controlled, n (%) (scores <15) N=83	p-value
**Salbutamol inhaler**				
Yes (n=100)	51 (16.6)	30 (18.4)	19 (20.9)	0.42
**Inhaled corticosteroids**				
Yes (37)	22 (7.2)	9 (5.5)	6 (7.2)	0.78
**Salbutamol tablets**				
Yes (96)	45 (14.7)	29 (17.8)	22 (26.5)	0.04
**Steroid tablets** (mostly prednisolone)				
Yes (149)	86 (28.1)	39 (23.9)	24 (28.9)	0.57
**Neither salbutamol nor steroids**				
Yes (225)	153 (49.8)	50 (30.7)	22 (26.8)	<0.0001
**Other medications ^§^**				
Yes (121)	68 (22.2)	32 (19.6)	21 (25.3)	0.59

ACT= Asthma Control Test; cACT=childhood Asthma Control Test; N=number; 2 ^nd^-4 ^th^ columns show numbers (percentages). ^§^Other medications mainly included aminophylline (tablets or injection), antihistamines, antibiotics, various cough and herbal remedies. Only three reported nebulised salbutamol. None reported leukotriene modifiers (such as montelukast) or antimalarials.

### Reported previous asthma assessments

We also enquired about previous asthma assessments and follow-up, and found that only 45 (8.2%) children had ever had a lung function test; only two (0.4%) had ever used a peak flow meter to monitor their asthma at home; 13.2% reported visiting a health facility to monitor their asthma; and only three (0.5%) had a personal written asthma action plan.

### Factors associated with having ever used inhaled asthma medication

We assessed factors associated with having ever used inhaled asthma medication, and found these were mother’s and father’s tertiary education (adjusted odds ratio [95% confidence interval]: 2.91 [1.61-5.25] and 5.19 [2.39-11.28], respectively); the child’s area of residence in early life (city residence at birth, 4.66 [1.79-12.43] or in the first five years, 2.99 [1.11-8.05]); and having ever had an asthma diagnosis (11.39 [6.35-20.43]) ( Table 4).

**Table 4.** Factors associated with having ever used inhaled asthma medication.

Characteristic	Ever used inhaled treatment? (N=553)		Adj. OR (95% CI) ^#^	p-value
	Yes n (%)	No n (%)		
**Mother’s education (m=6)**				
None/primary (n=154)	24 (15.6)	130 (84.4)	1	
Secondary (183)	43 (23.5)	140 (76.5)	1.26 (0.68-2.35)	
Tertiary (210)	81 (38.6)	129 (61.4)	2.91 (1.61-5.25)	0.0002
*P-value for trend*			*p<0.0001*	
**Father’s education (m=4)**				
None/primary (97)	10 (10.3)	87 (89.7)	1	
Secondary (189)	44 (23.3)	145 (76.7)	2.24 (1.00-5.02)	
Tertiary (263)	94 (35.7)	169 (64.3)	5.19 (2.39-11.28)	<0.0001
*P-value for trend*			*p<0.0001*	
**Area of residence at the time of the child’s birth**				
Rural (74)	9 (12.2)	65 (87.8)	1	
Town (411)	106 (25.8)	305 (74.2)	1.93 (0.86-4.35)	
City (68)	33 (48.5)	35 (51.5)	4.66 (1.79-12.43)	0.002
*P-value for trend*			*P=0.001*	
**Area of residence where child spent most of 0–5 years**				
Rural (71)	10 (14.1)	61 (85.9)	1	
Town (433)	114 (26.3)	319 (73.7)	1.60 (0.73-3.50)	
City (49)	24 (49.0)	25 (51.0)	2.99 (1.11-8.05)	0.08
*P-value for trend*			*p=0.03*	
**Ever had asthma diagnosis (m=7)**				
No (241)	17 (7.0)	224 (93.0)	1	
Yes (305)	131 (42.9)	174 (57.1)	11.39 (6.35-20.43)	<0.0001

N, number; Adj.OR, adjusted odds ratio; CI, confidence interval; m, missing; ^**#**^Adjusted for child’s age and sex, father’s education, area residence at birth, asthma diagnosis ever, and asthma control.

### Barriers to inhaled asthma medication use

We investigated why 405 of 553 (73%) children enrolled in this study had never used inhaled asthma medications, which are the mainstay of treatment including mild asthma ^16^. The reasons included that inhaled asthma medication had never been prescribed for them (47.6%, including children with a previous asthma diagnosis); no asthma diagnosis (38.8%); high cost of inhalers (4.5%); fear of side-effects of inhalers (4.5%); and alternative treatment with herbal or cough remedies (2.2%), or with salbutamol or steroid tablets (1.4%). Some children (1%) reported wrapping up in warm clothes or resting, without any medication.

### Reported asthma triggers, and treatment offered

The children reported the following, non-mutually exclusive, triggers for asthma: cold air (89.1%), chest infections (84.0%), physical exercise (78.0%), dust (66.8%), fumes or air pollution (61.9%), emotional distress or excitement (21.6%), pollen or pets (6.5%).

All children were offered one course of asthma treatment according to the Global Initiative for Asthma (GINA) 2015 treatment guidelines ^17^. This was determined by the study clinicians, using participants’ detailed asthma symptoms reported on the Asthma Control Test (ACT) or childhood Asthma Control Test (cACT). According to the GINA 2015 guidelines, 279 (50.4%) children received step I [salbutamol inhaler as needed], 209 (37.8%) children step II [salbutamol inhaler as needed and low dose inhaled corticosteroids], 52 (9.4%) children step III [salbutamol inhaler as needed, and low dose inhaled corticosteroids and long acting beta _2_-agonist], and 13 (2.4%) children step IV [salbutamol inhaler as needed, and medium dose inhaled corticosteroids and long acting beta _2_-agonist]. None received step V treatment. Participants were then referred to one of the two asthma clinics in the study area for further management, according to their preference.

## Discussion

We found that asthma control among Ugandan schoolchildren was poor, and this was mainly related to inadequate management. About half the children had either partly or poorly controlled asthma in the previous four weeks, 44% had asthma that had never been diagnosed, and only 7% had used the recommended inhaled corticosteroids in the last 12 months. These findings confirm that children with asthma in Uganda have poor asthma management, which stems from systemic failures of the health system.

The level of asthma control we observed was similar to that reported in a study from Cameroon ^18^. We found that poor asthma control was associated with three factors: increasing age, concurrent allergic rhinitis and city residence in early life. The association between concurrent allergic rhinitis and poor asthma control has been reported elsewhere ^19^. The association between city residence in early life and poor asthma control was particularly interesting. Our earlier work found that asthma risk among schoolchildren who resided in the city in their early years was three times higher than their classmates who resided in rural areas in early life ^6^. This suggests that city residence in early life is associated with both increased asthma risk and asthma severity.

Children with poor asthma control were less likely to engage in regular physical exercise. For some children, this was their way of controlling asthma symptoms. Other studies have observed that children with asthma symptoms avoid activities or exercise ^20, 21^. What was intriguing was the finding that children with poor asthma control were more likely to have received several doses of antimalarial treatment in the last year, yet antimalarials were not among medications that children reported using to control asthma symptoms. This is the first report of this kind. We did not ascertain whether these antimalarials were prescribed by medical workers or were self-administered. In Uganda, the antimalarials currently in use are artemisinin and its derivatives artesunate and artemether, which are either freely available from health service providers or easily available as over-the-counter medications (a positive laboratory test is not a requirement). It is important to investigate why poor asthma control was associated with increased use of antimalarials.

Asthma management in this urban area was very poor. Although inhaled medications (salbutamol and inhaled corticosteroids) are the main stay of asthma treatment ^16^, less than one-third of the children with asthma in this setting had ever been treated with any form of the recommended inhaled medication, and only 20% utilised inhaled salbutamol and 7% inhaled corticosteroids in the last 12 months. These percentages were no better for children with poor asthma control in the last four weeks. This suggests that the poor asthma control could be attributed to lack of correct asthma treatment. Instead, children with asthma were using inferior medications such as salbutamol tablets, which are known to cause numerous side-effects, and medications that have no role in asthma treatment, such as antihistamines, cough and herbal remedies. This was particularly disappointing because salbutamol and beclomethasone (an inhaled corticosteroid) inhalers are on the country’s Essential Medicines and Health Supplies list (EMHSLU) and in the National Treatment Guidelines.

Medical workers played an important role in the inadequate asthma management that we observed, from diagnosis and prescription to follow-up. Their inadequate prescription of inhaled asthma medications may have been related to the limited availability and affordability of inhaled asthma medications, as previously reported ^22^. However, their lack of accurate prescriptions could also have been related to their general lack of knowledge on current asthma management guidelines, which emphasise inhaled rather than oral formulations of salbutamol and steroids. Accurate asthma diagnoses were not always made. We noted that children with a prior asthma diagnosis had increased chances of having received inhaled asthma treatment, and that parents with tertiary education and those in the city were able to seek appropriate treatment for their children’s asthma. Therefore, there is an urgent need to re-train medical workers in asthma management in this setting, and to update the country’s National Treatment Guidelines to reflect the internationally recognised GINA guidelines, in which salbutamol tablets have no role in asthma treatment.

We recognise three main limitations of this study. First, because of the cross-sectional design, there was no follow-up of participants and therefore, we were not able to assess asthma severity. Asthma severity depends on the level of treatment required to control symptoms and exacerbations. Secondly, this study was not designed to collect data from health workers; therefore, we did not establish why health workers did not routinely prescribe inhaled asthma medications. Thirdly, we included only children with a history of wheezing and other asthma symptoms in the last 12 months, which may have excluded children with well-controlled asthma. This potential selection bias is likely to have introduced only minor imprecision to our findings.

The strength of this study was the large number of children with asthma recruited from the general population, thereby increasing the generalisability of the study findings. Our results suggest that the poor asthma management observed could be improved by: re-training health workers in asthma diagnosis and management; increasing patient and public education about asthma; increasing the availability and affordability of inhaled asthma medication; and updating the essential drugs list and national treatment guidelines to include the basic inhaled asthma medications recommended by the GINA treatment guidelines. Given the increasing prevalence of asthma among children in urban areas in LMICs, policy makers must prioritise asthma management at all levels of the health system. Medical training in the country needs to adapt to the epidemiological transition towards non-communicable diseases.

## Conclusion

This study confirms that several children in Uganda have inadequately controlled asthma, mostly because of lack of accurate diagnosis, treatment and follow-up.

## Data availability

### Underlying data

LSHTM Data Compass: SONA project - Asthma management data. https://doi.org/10.17037/DATA.00001358 ^14^.

This project contains the following underlying data:

- SONA_management_data.csv (This dataset includes all the variables that were used in this analysis. This includes participant demographics, previous asthma treatment and management history, asthma control and assessments at enrolment)
- Codebook.html (codebook for asthma management dataset)

### Extended data

LSHTM Data Compass: SONA project – Asthma management questionnaire: https://doi.org/10.17037/DATA.00001405 ^11^.

This project contains the following extended data:

- Asthma management questions (questionnaire in PDF and Microsoft Word format)

Data are available under the terms of the Creative Commons Attribution 3.0 International license (CC-BY 3.0).
