# Supplementary material for: Asthma control and management among schoolchildren in urban Uganda: results from a cross-sectional study
Source: Wellcome Open Res. 2019 Nov 5;4:168. [Version 1] doi: 10.12688/wellcomeopenres.15460.1 (PMC7327727; doi:10.12688/wellcomeopenres.15460.1)
Supplement: Peer review history

# Peer review: Reviewer response for version 1

**DOI:** 10.21956/wellcomeopenres.16907.r38829

**Published:** 2020-06-30

**Authors:** Pierluigi Paggiaro

**Affiliations:** 1 Department of Surgery, Medicine, Molecular Biology and Critical Care, University of Pisa, Pisa, Italy

**General comments**

 This paper reports an interesting survey performed in a large sample of children and adolescents in Uganda. The methodology used for selecting potential patients from a full sample of children at school is correct, and the authors were able to individuate a fairly large number of patients with suspected asthma, then confirmed with an appropriate assessment. In this way, the merit of this study was to have examined really patients with proven or very probable asthma. The results of the evaluation of these patients are in agreement with what was expected: poor asthma control, inappropriate previous diagnosis, very low use of anti-asthma drugs. Also the risk factors for poor asthma control are in line with previous known data (exposure to pollutants in inner city, patents’ education level, previous asthma diagnosis).

 The paper might be suitable, but it require a minor revision, mainly as regards some comments to be included in the discussion.

**Specific comments**

 Few considerations may required to be mentioned and commented on in the discussion

1. The socio-economic level of the participants seems fairly high, considering that 40% about of the parents reported a tertiary level of education. If so, the really low rate of prescription for asthma is more astonishing. It might be interesting to compare the present data with other previous surveys performed in other countries or in more poor or rural populations, to see if the data are similar.
2. Although 50% about of these children were affected by mild asthma, and therefore recommended to use salbutamol only as needed, according to GINA guidelines until 2018, the use of ICS was really very low, and also in more symptomatic patients only salbutamol plus oral steroids (probably in a short-course way) were used. This was completely in disagreement with all asthma guidelines
3. As regards the reason of the very low use of ICS, only 7% attributed this to a high cost of the drug (considering also the medium-high income of the studied population), but almost 50% reported a lack of prescription from their physician, also when asthma diagnosis was present. This suggest the need of a strong education plan of GPs, according to the implementation program of GINA.
4. What might be the reason for a so high use of salbutamol tablets ?

Is the work clearly and accurately presented and does it cite the current literature?

Yes

If applicable, is the statistical analysis and its interpretation appropriate?

Yes

Are all the source data underlying the results available to ensure full reproducibility?

Partly

Is the study design appropriate and is the work technically sound?

Yes

Are the conclusions drawn adequately supported by the results?

Partly

Are sufficient details of methods and analysis provided to allow replication by others?

Yes

Reviewer Expertise:

Asthma, severe asthma, biomarkers

I confirm that I have read this submission and believe that I have an appropriate level of expertise to confirm that it is of an acceptable scientific standard.

# Peer review: Reviewer response for version 1

**DOI:** 10.21956/wellcomeopenres.16907.r38389

**Published:** 2020-05-12

**Authors:** Inger Kull

**Affiliations:** 1 Institute of Environmental Medicine, Karolinska Institutet, Stockholm, Sweden

Thank you for the opportunity to review this paper aiming to asses the level of asthma control and management among children. Data arises from a cross sectional study among schoolchildren 5-17 years in Uganda. Asthma was measured by the validated asthma control test and the childhood Asthma control test.  The study covers a topic of high clinical importance for asthma management.

**Specific comments:**

 Abstract

 Conclusion: Data from the current study do indicate that the inadequate asthma control among a high proportion of children is due to poor asthma management. But can we be sure? I recommend that the authors rephrase the conclusion slightly.

**Background:**

 If possible, I suggest that you support the first statement in the section of background with a reference.

**Method:**

 The participants in the present study were recruited from a large case-control study. I suggest that you present the original cohort in a short sentence.

 The study population in the current consisted of 562 children from 55 schools. But how many were children were eligible at all schools? 562 children out of how many? n/N

**Results:**

 Interesting and clearly presented.

 A bit confusing the way the authors present the result,” adjusted odds ratio (95% Confidence interval), P-value and then the result. I suggest that you delete p-value, to read adjusted odds ratio, xxxx (95% CI xxx-xxx) p-value xxxx.

 An interesting result presented is that almost the same proportion reported ever used inhaled asthma medication and herbal remedies. Suggesting a gap between asthma guidelines and asthma management.

**Discussion**

 In the first paragraph of the discussion I expect to find the main result (as in the present version) but also information on study design.

 Conclusion:

 I find the conclusion at the end of the section preferable. Slightly different from the abstract.

 Minor comments:

 Key words: Please put key words in alphabetical order

 Abstract: A bit confusing the way the authors present the result,” adjusted odds ratio (95% Confidence interval), P-value and then the result. I suggest that you delete p-value, to read adjusted odds ration, xxxx (95% CI xxx-xxx) p-value xxxx

Is the work clearly and accurately presented and does it cite the current literature?

Yes

If applicable, is the statistical analysis and its interpretation appropriate?

Yes

Are all the source data underlying the results available to ensure full reproducibility?

No source data required

Is the study design appropriate and is the work technically sound?

Yes

Are the conclusions drawn adequately supported by the results?

Partly

Are sufficient details of methods and analysis provided to allow replication by others?

Partly

Reviewer Expertise:

Asthma management, epidemiology

I confirm that I have read this submission and believe that I have an appropriate level of expertise to confirm that it is of an acceptable scientific standard, however I have significant reservations, as outlined above.

# Peer review: Reviewer response for version 1

**DOI:** 10.21956/wellcomeopenres.16907.r37209

**Published:** 2019-12-10

**Authors:** Christopher Kuaban

**Affiliations:** 1 Faculty of Health Sciences, University of Bamenda, Bamenda, Cameroon

This is an interesting cross-sectional study whose objective was to assess the level of asthma control and management among school children aged 5 to 17 years with asthma in an urban area of Wakiso District in Uganda.

**Comments:**

- It is not quite clear in the methods what the definition used for designating a school child as asthmatic was. Was asthma defined using the ISAAC definition or it was “mostly clinical as a history of recurrent symptoms of wheezing, chest tightness, shortness of breath and cough that is mostly dry, worse at night and in the morning”? This should be made clear.
- By prescreening and screening for asthma only among children with “any current breathing problems”, it can be assumed that there was some selection bias as children with well controlled asthma might not have indicated that they had any current breathing problem. In this case the study might have recruited mostly children whose asthma was not well controlled. This could therefore lead to an over-estimation of poorly managed asthma in this population.
- It is important to indicate how many children were prescreened for the selection of the 562 or 561 children included in the study.
- It is observed that the proportion of children who had ever had a diagnosis of asthma is practically same as that of children whose asthma was well controlled (respectively 55.9% and 55.5%). Could this not mean that the majority of children with poor asthma control was mostly made up of children whose asthma was only diagnosed due to or during the study?
- The authors state that they “looked at the correlation between asthma control in the last four weeks and type of asthma medication used in the last 12 months to assess the proportion of children currently with poor asthma control who have received the correct asthma treatment at any point in the last 12 months”. The reason given for this analysis is not convincing as it is not clear how receiving correct medication at any point in the last 12 months can explain poor asthma control in the last four weeks. By the way, nowhere in the text is it mentioned that these children had persistent asthma to warrant that one should guess that they were prescribed daily maintenance therapy and that they might have abandoned treatment thereby causing poor control of their disease. This guess even cannot be true as “clinicians using participants’ detailed asthma symptoms” prescribed to 279 (50.4%) children step 1 treatment (Salbutamol as needed). By the way, do we speak of asthma control when patients have a crisis from time to time? I believe asthma control using the scales the authors used is indicated essentially for evaluating control in patients with persistent asthma under medication.
- It would have been interesting to find out if poor asthma control was related to barriers to inhaled asthma medication use and reported asthma triggers and even treatment offered for these triggers rather than just give proportions of these factors found in the children. This is important because apart from medication, preventive measures should be included as a strategy in the management of these patients.
- The discussion and abstract have not been reviewed because they might have to be modified in light of the comments above.

Is the work clearly and accurately presented and does it cite the current literature?

Yes

If applicable, is the statistical analysis and its interpretation appropriate?

I cannot comment. A qualified statistician is required.

Are all the source data underlying the results available to ensure full reproducibility?

Partly

Is the study design appropriate and is the work technically sound?

Partly

Are the conclusions drawn adequately supported by the results?

Partly

Are sufficient details of methods and analysis provided to allow replication by others?

Partly

Reviewer Expertise:

Respiratory disorders

I confirm that I have read this submission and believe that I have an appropriate level of expertise to confirm that it is of an acceptable scientific standard, however I have significant reservations, as outlined above.
